# Prevalence and determinants of diarrhoea among children under five years: A pooled facility-based cross-sectional study across three ecological zones in Ghana

**DOI:** 10.1371/journal.pone.0361693

**Published:** 2026-11-05

**Authors:** Anthony Zunuo Dongdem, Edmond Datiekah Dordaah, Geraldo Elikem Lily, Patience Akyeah, Prince Emmanuel Kwamena Amissah, Doreen Selasie Tay, Patience Abla Ayiglo, James Okofo Asenso

**Affiliations:** 1 Department of Epidemiology and Biostatistics, Fred N. Binka School of Public Health, University of Health and Allied Sciences, Ho, Ghana; 2 Jirapa Municipal Health Directorate, Ghana Health Service, Jirapa, Ghana; 3 Ga West Municipal Health Directorate, Ghana Health Service, Amasaman, Ghana; 4 Gomoa East District Health Directorate, Ghana Health Service, Buduatta, Ghana; Kintampo Health Research Centre, GHANA

## Abstract

**Background:**

Diarrhoea remains a major cause of morbidity and mortality among children under five years in sub-Saharan Africa. Although national estimates are available in Ghana, district-level evidence across different ecological settings is limited. This study estimated the prevalence and determinants of diarrhoea among children under five across three geographically diverse districts in Ghana.

**Methods:**

A pooled analysis of three facility-based cross-sectional studies was conducted in the Ga West Municipality, Gomoa East District, and Jirapa Municipal District of Ghana. A total of 1,266 caregiver–child pairs (422 per district) were selected using stratified random sampling. Data were collected through structured interviewer-administered questionnaires. The primary outcome was caregiver-reported diarrhoea within six months preceding the survey. Multivariable logistic regression was performed to identify independent determinants of diarrhoea at both district and pooled levels using STATA version 17.

**Results:**

The pooled prevalence of diarrhoea was 66.4% (95% CI: 63.7–69.0), with marked district variations: 91.7% in Ga West, 72.3% in Gomoa East, and 34.8% in Jirapa.In the pooled multivariable model, children in Gomoa East (AOR = 0.24; 95% CI: 0.12–0.45) and Jirapa (AOR = 0.07; 95% CI: 0.03–0.13) had significantly lower odds of diarrhoea than those in Ga West. Children aged 9–16 months (AOR = 3.47; 95% CI: 1.69–5.47), 17–25 months (AOR = 5.07; 95% CI: 3.05–8.44), and 26–59 months (AOR = 3.63; 95% CI: 2.14–6.14) had higher odds of diarrhoea than infants aged 1–8 months, and unimproved water source was independently associated with increased odds (AOR = 1.99; 95% CI: 1.19–3.36). Protective factors included tertiary education (AOR = 0.33; 95% CI: 0.19–0.57), health insurance enrolment (AOR = 0.60; 95% CI: 0.37–0.95), and availability of handwashing facilities (AOR = 0.53; 95% CI: 0.38–0.74), while caregiver knowledge that handwashing prevents diarrhoea (AOR = 3.44; 95% CI: 1.81–6.54) and rotavirus vaccination (AOR = 3.04; 95% CI: 1.69–5.47) were paradoxically associated with higher odds.

**Conclusions:**

Diarrhoea burden among children under five remains high and varies considerably across districts in Ghana. Strengthening water, sanitation, and hygiene interventions and targeting vulnerable age groups, and addressing underlying district-level drivers may help reduce the burden of childhood diarrhoea.

## Introduction

Diarrhoea is defined as the passage of three or more loose or liquid stools within a 24-hour period and is commonly a symptom of gastrointestinal infection caused by viral, bacterial, or parasitic pathogens [1]. Globally, diarrhoeal disease remains a leading cause of morbidity and mortality among children under five, accounting for an estimated 1.7 billion cases annually [1]. Inadequate water, sanitation, and hygiene (WASH) infrastructure, unsafe drinking water, poor maternal education, and poverty remain key drivers of diarrhoeal transmission in low- and middle-income countries [2]. Sub-Saharan Africa bears a disproportionate share of this burden due to persistent inequalities in sanitation coverage and healthcare access. A recent systematic review in East Africa reported a pooled under-five diarrhoea prevalence of 24.6% and identified household sanitation, water source, and socioeconomic status as major determinants [3]. Despite global progress in oral rehydration therapy, rotavirus vaccination, and WASH interventions, diarrhoeal disease continues to undermine child survival gains in many African settings.

In Ghana, diarrhoeal disease remains a significant public health concern among children under five. The 2022 Ghana Demographic and Health Survey reported a two-week prevalence of approximately 13%, with notable disparities across regions and socioeconomic groups [4]. Diarrhoea contributes substantially to outpatient attendance and childhood hospital admissions and remains closely associated with environmental sanitation, maternal education, and household wealth status. Recent studies in coastal and urban Ghanaian communities further highlight the role of water quality, sanitation practices, and climatic factors such as rainfall and flooding in shaping disease patterns [5]. Kombat and Kushitor documented a declining national trend from 26% in 1988 to 13% in 2022, attributing the reduction to improvements in WASH conditions, breastfeeding promotion, and expanded vaccination coverage, though substantial subnational disparities persist [6].

The analytical child survival framework by Mosley and Chen provides a useful conceptual lens for this study [7]. The framework posits that all social and economic determinants of child morbidity and mortality operate through a common set of biological and proximate mechanisms. Within this framework, child-level factors (age, sex, vaccination status), household-level factors (water source, sanitation facilities, income, education), and community-level factors (residential setting, ethnicity, NHIS access) are conceptualised as operating along a causal pathway to produce the observed diarrhoeal burden. This framework guided the selection and organisation of independent variables in the present study.

Although several district-level studies have examined diarrhoea among children under five in Ghana, most are geographically restricted, limiting the generalisability of findings across diverse ecological and sociodemographic contexts. The three districts included in this study, Jirapa Municipal, Ga West Municipality, and Gomoa East District, represent varied environmental and socioeconomic conditions that may influence diarrhoeal burden differently. However, evidence synthesising findings across such heterogeneous districts using comparable methodologies remains limited.

This study, therefore, aimed to estimate the district-specific and overall pooled prevalence of diarrhoea among children under five in Jirapa, Ga West, and Gomoa East, and identify the independent determinants of diarrhoea at the district and pooled levels. By integrating data from three independent cross-sectional studies, this pooled analysis provides more comprehensive evidence to inform targeted child health and WASH interventions in Ghana. Specifically, the objectives of this study were to: (1) estimate the district-specific and overall pooled prevalence of diarrhoea among children under five in Jirapa, Ga West, and Gomoa East: and (2) identify the independent determinants of diarrhoea at both the district and pooled levels.

## Materials and methods

### Study design and setting

This was a facility-based cross-sectional study conducted from May to August 2024, among caregivers of children under five years in three districts of Ghana: Jirapa Municipal (Upper West Region), Ga West Municipality (Greater Accra Region), and Gomoa East District (Central Region). The study aimed to estimate the prevalence of diarrhoea among children under five and identify associated determinants. The study was conducted and reported in accordance with the Strengthening the Reporting of Observational Studies in Epidemiology (STROBE) guidelines for cross-sectional studies.

Jirapa Municipal is predominantly rural, characterised by subsistence agriculture and limited access to improved water and sanitation infrastructure. Ga West Municipality is largely peri-urban with rapid population growth and sanitation challenges linked to urban expansion. Gomoa East District comprises both rural and semi-urban communities with mixed livelihood systems and varying access to healthcare and WASH services. These ecological and sociodemographic differences provide diverse contexts for examining diarrhoeal burden across the three sites. Jirapa Municipal lies in the north-western corner of the Upper West Region, bordered by the Lambussie-Karni, Nadowli-Kaleo, Lawra, and Sissala West districts, and covers approximately 1,189 km2 of predominantly savanna landscape supporting subsistence farming of cereals, legumes, shea, cotton, and ground nuts. Ga West Municipality lies within the Greater Accra Region on the north-western fringe of Accra, bordered by the Ga North, Ga South and Ga Central, and covers about 300 km2 of rapidly urbanising land where sachet and piped water are the dominant drinking-water sources. Gomoa East District lies in the south-eastern Central Region, bordered by Agona East, Gomoa West, Awutu Senya, Ga South, and Effutu, and covers approximately 540 km2 comprising both coastal/semi-urban settlements and rural farming communities [8–11]. These geographic and infrastructural differences underpin the selection of the three districts as proxies for Ghana’s savanna, coastal-urban, and mixed rural-semi-urban ecological zones, respectively (Fig 1).

**Fig 1:**
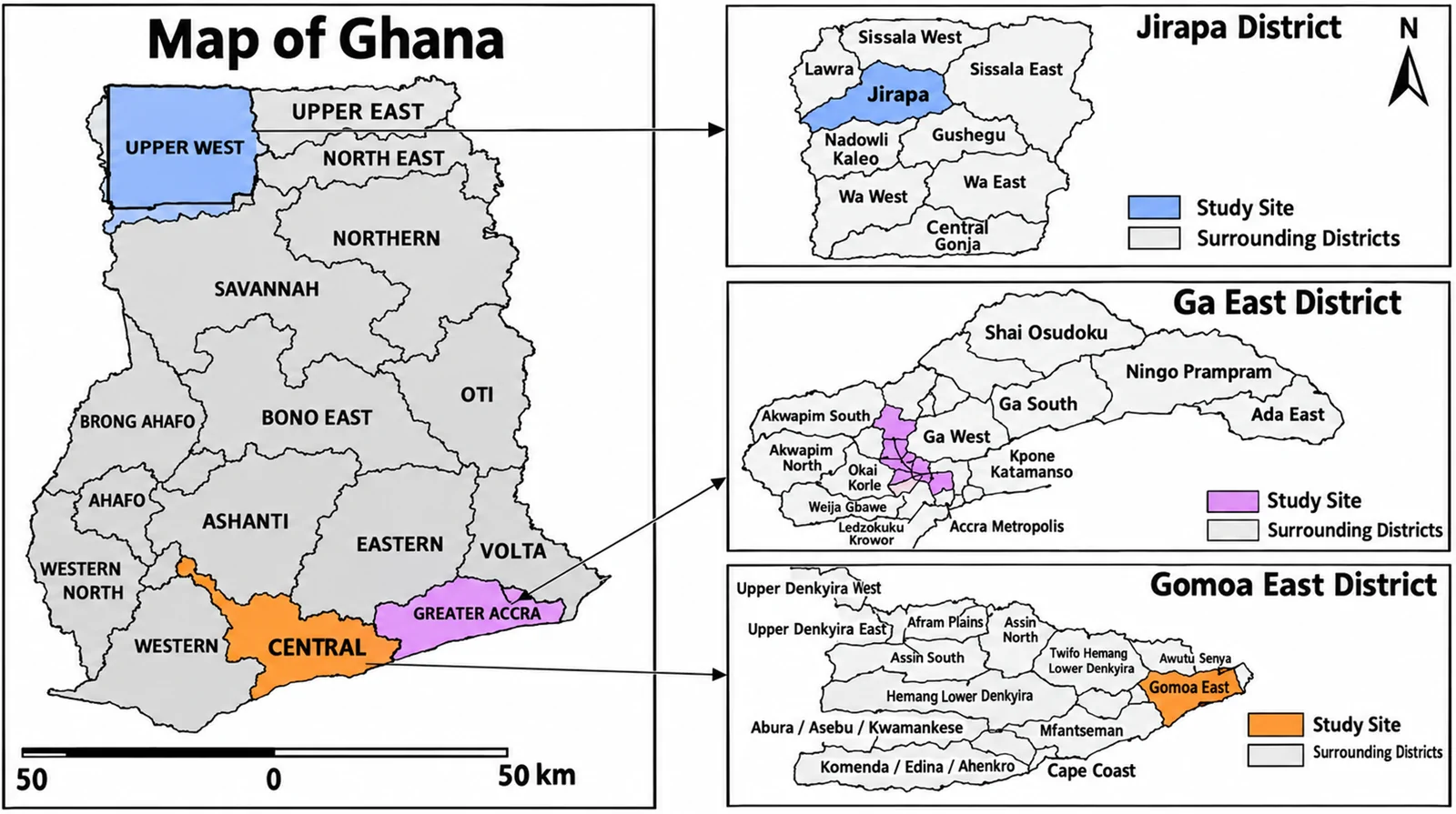
Study districts in Ghana (Administrative boundary shapefiles were obtained from the Database of Global Administrative Areas (GADM, version 4.1), and the map was prepared using ArcGIS).

### Study population and eligibility

The study population comprised caregivers (primarily mothers) of children aged 0–59 months who attended selected health facilities within the three districts during the study period. Caregivers who gave informed consent and had resided in the district for at least six months were included. Children with severe illness preventing meaningful caregiver participation were excluded. Caregivers were identified consecutively as they attended child welfare clinic sessions at each selected facility until the proportionate sample size for that facility was reached; those who declined to participate or could not complete the interview (for example, because of a language barrier not resolved by the trained interpreters) were also excluded and replaced with the next eligible caregiver. Completed questionnaires were checked for completeness and internal consistency by field supervisors on the day of collection, and any forms with missing or contradictory responses were returned to the data collector for follow-up with the respondent before entry, in order to minimise missing data and improve reproducibility.

### Sample size

The sample size for each of the three district-level studies was determined using Cochran’s formula for cross-sectional prevalence studies: n = Z²p(1-p)/d², where Z = 1.96 (95% confidence level), p = 0.50 (assumed prevalence in the absence of district-specific prior estimates), and d = 0.05 (margin of error) [12]. This yielded a minimum sample size of 384, to which a 10% non-response allowance was added, giving a final target of 422 participants per district. For the present pooled analysis, the three datasets were combined to form a single analytical dataset of 1,266 participants, increasing statistical power and enabling both district-specific and pooled estimation.

### Sampling procedure

A two-stage stratified sampling procedure was employed. In the first stage, each district was stratified by sub-district, and two sub-districts were selected using simply random sampling by the lottery method. All health facilities and Community-based Health Planning and Services (CHPS) zones within the two selected sub-districts were included in the study. The selected facilities were St. Joseph Hospital, Tizza Health Centre, Duori Health Centre, and Jirapa Urban Health Centre in Jirapa Municipality; Akramaman Health Centre, Obeyeyie Health Centre, Oduman Polyclinic, and Joma Health Centre in Ga West Municipality; and Ojobi Health Centre, Buduatta Health Centre, Hope Christian Hospital, St. Gregory Hospital, Fetteh CHPS Compound, and Akraman CHPS Compound in Gomoa East District.

In the second stage, caregivers were randomly selected from the included health facilities and CHPS zones using the lottery technique until the required sample was attained. The number of caregivers selected from each sub-district was proportional to its population size, ensuring representativeness across sub-districts.

### Variables

The primary outcome variable was diarrhoea occurrence among children under five, defined as caregiver-reported passage of three or more loose or watery stools within a 24-hour period at any point during the six months preceding the survey. A six-month recall period was used, rather than the standard two-week WHO surveillance recall, in order to capture seasonal variation in diarrhoeal burden across the study districts and to detect cumulative illness episodes more reflective of the local disease context. The outcome variable was coded as 1 = presence of diarrhoea and 0 = no diarrhoea. Independent variables were harmonised across the three datasets and included: child characteristics (age, sex, vaccination status, deworming history); caregiver characteristics (age, marital status, educational level, monthly income); and household and environmental characteristics (water source, latrine usage, handwashing facilities, domestic animals, NHIS enrolment, family size, ethnicity, and area of residence).

### Data collection

Data were collected using structured interviewer-administered questionnaires adapted from validated tools, including the Ghana Demographic and Health Survey framework. The questionnaires captured sociodemographic characteristics, environmental conditions, hygiene practices, health insurance status, and diarrhoeal episodes. Trained data collectors conducted face-to-face interviews at selected health facilities after obtaining written informed consent. Supervisors ensured data quality through daily review of completed questionnaires and on-site monitoring.

### Data management and analysis

Data from the three studies were cleaned, entered, and analysed using STATA version 17. For the pooled analysis, datasets were harmonised by aligning variable definitions, recoding categories, and ensuring consistency in outcome measurement across all three datasets. Descriptive statistics, including frequencies and proportions for categorical variables and means and standard deviations for continuous variables, were used to summarise participant characteristics. Water source was categorised as improved or unimproved based on WHO/UNICEF Joint Monitoring Programme criteria [13].

Prevalence was calculated as the proportion of children reported to have experienced diarrhoea during the preceding six months. An overall pooled prevalence was estimated without population weighting, given that each district contributed an equal sample size of 422 participants, and the study objective was to compare district-level burden rather than produce population-representative national estimates. It should therefore be noted that unweighted pooling may not fully account for differences in the population sizes represented by each district.

Bivariate logistic regression was conducted to assess crude associations between independent variables and diarrhoea occurrence. All variables with p < 0.25 at bivariate level were entered into the multivariable logistic regression model. Multivariable logistic regression was performed for each district independently and for the pooled dataset to identify independent determinants of diarrhoea. Results are reported as crude odds ratios (COR) and adjusted odds ratios (AOR) with 95% confidence intervals. Statistical significance was set at p < 0.05.

### Ethical considerations

Ethical approval was obtained from the University of Health and Allied Sciences, Ethical Review Committee, protocol number (UHAS-REC B.10[038]23–24), on 10th April 2024. Permission was obtained from the district health directorates and selected health facilities. Written informed consent was obtained from all participating caregivers (parents or guardians). For caregivers who were minors, assent was obtained prior to data collection, in addition to consent from their parent or legal guardian before data collection. Confidentiality was maintained by anonymising questionnaires and restricting data access to the research team. Participation was voluntary, and respondents were free to withdraw at any stage without consequence.

## Results

### Socio-demographic characteristics of participants

A total of 1,266 caregivers and their children under five years were recruited, with 422 participants per district. All participants in Ga West were from urban areas, while the majority in Gomoa East were rural dwellers (86.73%). Jirapa had a mixed composition, with 61.85% urban and 38.15% rural residents. Most caregivers in Ga West (64.45%) and Jirapa (85.07%) were married, while most in Gomoa East were single (72.51%). Tertiary education was the most common educational level across all three districts, accounting for 31.28%, 34.12%, and 26.54% in Ga West, Gomoa East, and Jirapa, respectively. The predominant ethnic groups were Ga/Dangme in Ga West, Akan in Gomoa East, and Mole-Dagbani in Jirapa. The majority of caregivers earned less than Ghana cedis (GH₵) 1,000 per month, and most held valid National Health Insurance Scheme (NHIS) cards. Rotavirus vaccination coverage was high across all districts, exceeding 91%. The proportion of children who experienced diarrhoea in the preceding six months was highest in Ga West (91.71%) and lowest in Jirapa (34.83%). Further details of participant characteristics are presented in Table 1.

**Table 1:** Sociodemographic and household characteristics of participants by district.

Variable	Districts		
**Area of residence**	**Ga West** **n (%)**	**Gomoa East** **n (%)**	**Jirapa** **n (%)**
Urban	422(100.00)	56(13.27)	261(61.85)
Rural	0(0.00)	366(86.73)	161(38.15)
**Age of caregiver**			
15-25	131(31.04)	77(18.25)	139(32.94)
26-30	109(25.83)	92(21.80)	106(25.12)
31-35	97(22.99)	121(28.67)	104(24.64)
36-66	85(20.14)	132(31.28)	73(17.30)
**Marital Status**			
Divorced	2(0.47)	22(5.21)	8(1.90)
Married	272(64.45)	84(19.91)	359(85.07)
Single	146(34.60)	306(72.51)	51(12.09)
Widowed	2(0.47)	10(2.37)	4(0.95)
**Educational Level**			
JHS	68(16.11)	128(30.33)	66(15.64)
No formal Education	45(10.66)	71(16.82)	104(24.64)
Primary	83(19.67)	30(7.11)	67(15.88)
SHS	94(22.27)	49(11.61)	73(17.30)
Tertiary	132(31.28)	144(34.12)	112(26.54)
**Ethnicity**			
Akan	115(27.25)	200(47.39)	31(7.35)
Ewe	96(22.75)	102(24.17)	10(2.37)
Ga\Dangme	141(33.41)	107(25.36)	18(4.27)
Mole-Dagbani	45(10.66)	11(2.61)	360(85.31)
Others	25(5.95)	2(0.47)	3(0.71)
**Monthly Income (GH₵)**			
Greater than 2000	50(11.85)	120(28.44)	15(3.55)
1500-2000	173(41.00)	135(31.99)	125(29.62)
Less than 1000	196(47.15)	167(39.57)	282(66.82)
**Health Insurance**			
No	43(10.19)	42(9.95)	87(20.62)
Yes	379(89.81)	380(90.05)	335(79.38)
**Family Size**			
1-2	193(45.73)	35(8.29)	234(55.98)
3-4	181(42.89)	267(63.27)	164(39.23)
5 or more	48(11.37)	120(28.44)	20(4.78)
**Rota Virus Vaccination for Child**			
No	23(5.45)	36(8.53)	33(7.82)
Yes	399(94.55)	386(91.47)	389(92.18)
**Child diarrhoea experienced for the past 6 months**			
No	35(8.29)	116(27.49)	275(65.17)
Yes	387(91.71)	306(72.51)	147(34.83)
**Age of Child (months)**			
1-8	138(32.70)	113(26.78)	68(16.11)
9-16	159(37.68)	66(15.64)	123(29.15)
17-25	118(27.96)	79(18.72)	88(20.85)
26-89	7(1.66)	164(38.86)	143(33.89)
**Sex of Child**			
Female	197(46.68)	195(46.21)	182(43.13)
Male	225(53.32)	227(53.79)	240(56.87)
**Child received dewormer at 24 months and above**			
No	383(90.76)	273(64.69)	145(34.36)
Yes	39(9.24)	149(35.31)	277(65.64)
**Water source**			
Improved	293(69.43)	302(71.56)	410(97.16)
Unimproved	129(30.57)	120(28.44)	12(2.84)
**Latrine Usage in the house**			
No	48(11.37)	25(5.92)	108(25.59)
Yes	374(88.63)	397(94.08)	314(74.41)
**Domestic animal present in the house**			
No	109(25.83)	18(4.27)	26(6.16)
Yes	313(74.17)	404(95.73)	396(93.84)
**Washing hands before breastfeeding/cooking**			
No	60(14.22)	9(2.13)	124(29.38)
Yes	362(85.78)	413(97.87)	298(70.62)
**Availability of handwashing facilities in the house**			
No	254(60.19)	225(53.32)	176(41.90)
Yes	168(39.81)	197(46.68)	244(58.10)
**Handwashing with soap help prevent diarrhoea infection**			
No	15(3.55)	3(0.71)	122(28.91)
Yes	407(96.45)	419(99.29)	300(71.09)
**Diarrhoea transmission through contaminated water**			
No	34(8.06)	6(1.42)	153(36.26)
Yes	388(91.94)	416(98.58)	269(63.74)
**Diarrhoea transmission through contaminated food**			
No	31(7.35)	7(1.66)	117(27.79)
Yes	391(92.65)	415(98.34)	304(72.21)

Values are presented as n (%) unless otherwise stated; percentages are calculated within each district column. Age of caregiver and age of child are reported in years and months, respectively; monthly income is reported in Ghana cedis.

### Diarrhoea prevalence

The overall pooled prevalence of diarrhoea among children under five years across the three districts was 66.35% (95% CI: 63.67–68.95). At the district level, Ga West recorded the highest prevalence at 91.70% (95% CI: 88.65–94.15), followed by Gomoa East at 72.25% (95% CI: 67.99–76.71), while Jirapa recorded the lowest prevalence of 34.83% (95% CI: 30.29–39.59). These findings are presented in Table 2 and illustrated in Fig 2, which shows the overall diarrhoea prevalence across the three districts.

**Table 2:** Diarrhoea Prevalence Among Children Under Five by District.

District	Diarrhoea experienced for the past 6 months		Total	Prevalence (%) (95% conf. interval)
	No	Yes		
Ga West	35	387	422	91.70(88.65-94.15)
Gomoa East	116	306	422	72.25(67.99-76.71)
Jirapa	275	147	422	34.83(30.29-39.59)
Overall pooled prevalence	426	840	1266	66.35(63.67-68.95)

“No” and “Yes” refer to whether the child experienced diarrhoea in the six months preceding the survey; prevalence (%) is the proportion of “Yes” out of the total, with 95% Wilson confidence intervals.

**Fig 2:**
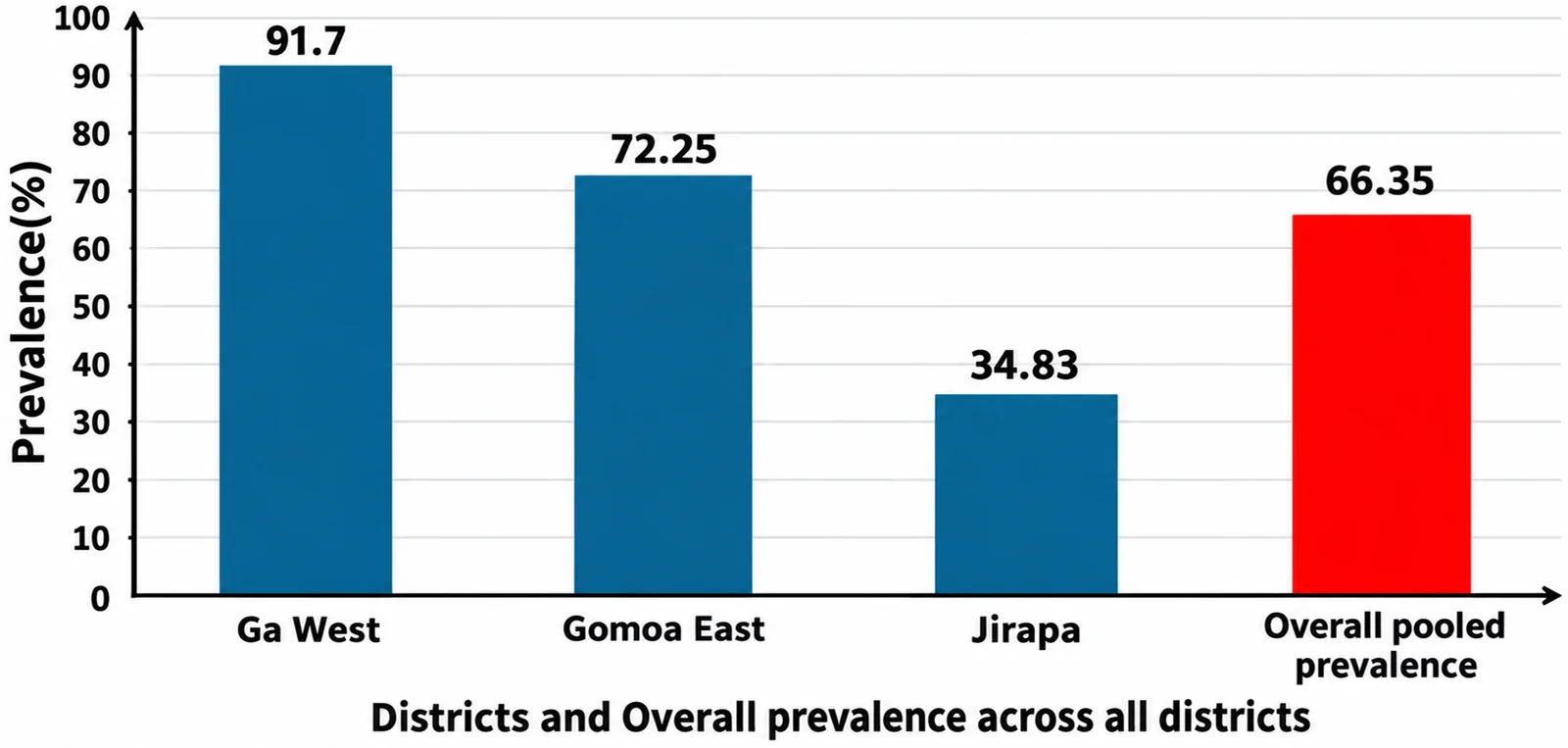
Overall diarrhoea prevalence across three districts.

### Determinants of diarrhoea: Bivariate and multivariable logistic regression

Bivariate analysis identified several variables significantly associated with diarrhoea occurrence at p < 0.25 across all three districts, which were subsequently entered into multivariable logistic regression models.

In the district-specific multivariable analysis, child age was a consistent and significant predictor across all three sites. In Ga West, children aged 9–16 months (adjusted odds ratio [AOR]: 8.46; 95% CI: 2.95–24.22; p < 0.001) and 17–25 months (AOR: 11.40; 95% CI: 2.46–52.84; p = 0.002) had markedly higher odds of diarrhoea compared to infants aged 1–8 months. Unimproved water source was associated with 5.59-fold higher odds (AOR: 5.59; 95% CI: 1.33–23.33; p = 0.018), while NHIS enrolment reduced odds by 62.4% (AOR: 0.376; 95% CI: 0.15–0.94; p = 0.037) and availability of handwashing facilities reduced odds by 67.1% (AOR: 0.329; 95% CI: 0.12–0.87; p = 0.025). In Gomoa East, children aged 17–25 months and 26–89 months had over tenfold higher odds of diarrhoea (AOR: 10.23; 95% CI: 3.62–28.84; p < 0.001 and AOR: 11.20; 95% CI: 3.90–32.10; p < 0.001, respectively). Presence of domestic animals in the household (AOR: 7.54; 95% CI: 1.10–51.50; p = 0.039), Ga/Dangme ethnicity (AOR: 2.75; 95% CI: 1.30–5.83; p = 0.008), and rotavirus vaccination (AOR: 9.52; 95% CI: 2.88–31.47; p = 0.026) were independently associated with increased odds, whereas tertiary education was associated with an 85.9% reduction in odds (AOR: 0.141; 95% CI: 0.03–0.51; p = 0.003). In Jirapa, unimproved water source was the strongest predictor, associated with over tenfold higher odds (AOR: 10.14; 95% CI: 2.06–49.92; p = 0.004). Caregiver age 31–35 years (AOR: 2.26; 95% CI: 1.02–5.02; p = 0.045) and child age 17–25 months (AOR: 2.56; 95% CI: 1.10–6.03; p = 0.029) were also significant independent risk factors. Latrine use reduced odds by 60.8% (AOR: 0.392; 95% CI: 0.21–0.73; p = 0.003), NHIS enrolment by 52.9% (AOR: 0.471; 95% CI: 0.26–0.85; p = 0.012), and single marital status by 66.0% (AOR: 0.34; 95% CI: 0.13–0.92; p = 0.034).

In the pooled multivariable model. Several factors were independently associated with diarrhoea occurrence after adjusting for cofounders (Table 3). Compared with Ga West, children in Gomoa East (AOR: 0.236; 95% CI: 0.12–0.45; p < 0.001) and Jirapa (AOR: 0.068; 95% CI: 0.03–0.13; p < 0.001) had substantially lower odds of diarrhoea. Tertiary education emerged as a protective factor (AOR: 0.33; 95% CI: 0.19–0.57; p < 0.001), as did health insurance (NHIS) enrolment (AOR: 0.60; 95% CI: 0.37–0.95; p = 0.033) and availability of household handwashing facilities (AOR: 0.53; 95% CI: 0.38–0.74; p < 0.001). Compared to children aged 1–8 months, those aged 9–16 months (AOR: 3.47; 95% CI: 1.69–5.47; p < 0.001), 17–25 months (AOR: 5.07; 95% CI: 3.05–8.44; p < 0.001), and 26–89 months (AOR: 3.63; 95% CI: 2.14–6.14; p < 0.001) had significantly higher odds of diarrhoea, and unimproved water source was found to be an independent risk factor (AOR: 1.99; 95% CI: 1.19–3.36; p = 0.009). Caregiver age 31–35 years, relative to 15–25 years, was independently associated with higher odds (AOR: 1.69; 95% CI: 1.05–2.72; p = 0.032). Caregiver knowledge that handwashing with soap prevents diarrhoea were paradoxically associated with higher odds (AOR: 3.44; 95% CI: 1.81–6.54; p < 0.001), as did rotavirus vaccination (AOR: 3.04; 95% CI: 1.69–5.47; p < 0.001), a finding that continues to warrant cautious interpretation given the facility-based design of the study.

**Table 3:** Bivariate and multivariate logistic regression between diarrhoea among children under five within the past six months and its associated factors.

Variable	COR (95% conf. interval) p-value				AOR (95% conf. interval) p-value			
	Ga West	Gomoa East	Jirapa	Overall pooled data	Ga West	Gomoa East	Jirapa	Overall pooled data
**District**								
Ga West				Ref				Ref
Gomoa East				**0.239(0.16-0.36)0.000**				**0.236(0.12-0.45)0.000**
Jirapa				**0.048(0.03-0.07)0.000**				**0.0678(0.03-0.13)0.000**
**Area of residence**								
Urban	Ref	Ref	Ref	Ref		Ref	Ref	Ref
Rural	1	**0.608(0.30-1.22)0.161**	**0.550(0.36-0.84)0.006**	**0516(0.40-0.65)0.000**		1.237(0.38-4.03)0.725	0.890(0.48-1.64)0.712	0.853(0.54-1.34)0.492
**Age of guardian**								
15-25	Ref	Ref	Ref	Ref			Ref	Ref
26-30	0.610(0.26-1.45)0.264	0.946(0.50-1.80)0.87	**2.026(1.66-3.52)0.012**	**1.243(0.91-1.71)0.178**			1.769(0.82-3.81)0.144	1.562(0.98-2.49)0.059
31-35	1.520(0.50-4.60)0.458	0.973(0.53-1.79)0.931	**2.548(1.47-4.42)0.001**	**1.456(1.06-1.99)0.020**			**2.261(1.02-5.02)0.045**	**1.688 (1.05-2.72)0.032**
36-66	0.920(0.34-2.52)0.857	2.692(1.37-5.28)0.004	**1.885(1.02-3.49)0.043**	**2.035(1.45-2.86)0.000**			1.678(0.68-4.20)0.269	1.493(0.87-2.55) 0.143
**Marital Status**								
Marrried	Ref	Ref	Ref	Ref	Ref		Ref	Ref
Divorced	**1**	**5(1.09-22.92)0.038**	1.699(0.42-6.90)0.456	**2.772(1.23-6.82)0.026**	1		2.429(0.42-14.06)0.322	2.128(0.66-6.91)0.209
Single	1.540(0.70-3.39)0.284	1.28(0.76-2.15)0.351	**0.316(0.14-0.69)0.004**	**1.693(1.32-2.17)0.000**	1.444(0.53-3.94)0.473		**0.34(0.13-0.92)0.034**	0.859(00.57-1.30)0.476
Widowed	**0.101(0.00-1.67)0.109**	1	1.699(0.24-12.20)0.598	**2.77(0.78-9.81)0.114**	**0.035(0.00-0.75)0.032**		3.31(0.36-30.25)0.289	2.127(0.42-10.80)0.362
**Educational Level**								
No formal Education	Ref	Ref	Ref	Ref	Ref	Ref	Ref	Ref
JHS	0.566(0.17-1.93)0.363	0.655(0.30-1.41)0.279	0.711(0.38-1.33)0.289	0.711(0.38-1.33)0.289	0.556(0.53-3.94)0.473	0.512(0.19-1.36)0.179	1.414(0.59-3.37)0.434	0.732(0.43-1.24)0.249
Primary	**3.951(0.69-22.47)0.121**	1.65(0.43-6.39)0.469	**0.651(0.35-1.22)0.183**	**0.651(0.35-1.22)0.183**	3.212(0.42-24.81)0.263	0.959(0.18-5.22)0.961	0.870(0.39-1.95)0.736	1.012(0.56-1.84)0.967
SHS	1.737(0.44-6.81)0.428	1.314(0.45-3.83)0.617	**0.645(0.35-1.19)0.163**	**0.645(0.35-1.19)0.163**	3.439(0.63-18.65)0.263	1.059(0.29-3.76)0.929	1.275(0.53-3.04)0.584	1.046(0.59-1.85)0.877
Tertiary	0.822(0.266-2.64)0.742	**0.205(0.09-0.42)0.000**	**0.318(0.18-0.58)0.000**	**0.318(0.18-0.58)0.00**	1.600(0.35-7.37)0.547	**0.141(0.03-0.51)0.003**	0.564(0.22-1.43)0.227	**0.333(0.19-0.57)0.000**
**Ethnicity**								
Akan	Ref	Ref	Ref	Ref		Ref		Ref
Ewe	0.819(0.33-2.06)0.671	1.129(0.67-1.89)0.646	0.9(0.89-0.4.23)0.894	**1.297(0.87-1.94)0.205**		1.67(0.83-3.39)0.153		1.211(0.74-1.98)0.446
Ga\Dangme	1.13(0.46-2.75)0.795	**2.676(1.26-4.92)0.002**	1.34(0.40-4.48)0.639	**2.271(1.50-3.45)0.000**		**2.750(1.30-5.83)0.008**		1.659(1.00-2.74)0.049
Mole-Dagbani	2.048(0.43-9.74)0.368	**0.392(0.12-1.33)0.134**	1.103(0.50-2.42)0.806	**0.267(0.20-0.36)0.000**		0.461(0.07-3.07)0.423		0.760(0.43-1.35)0.356
Others	1.095(0.22-5.34)0.910	1	1	**5.299(1.24-22.68)0.025**		1		1.562(0.27-9.05)0.619
**Monthly Income (GH₵)**								
Greater than 2000	Ref	Ref	Ref	Ref		Ref		
1500-2000	1.490(0.50-4.45)0.475	**1.990(1.16-3.40)0.012**	0.809(0.26-2.53)0.716	1.124(0.78-1.62)0.530		1.774(0.66-3.39)0.252		
Less than 1000	1.117(00.39-3.17)0.835	**2.342(1.39-3.94)0.001**	1.205(0.40-3.62)0.740	0.916(0.65-1.29)0.619		0.840(0.36-1.94)0.685		
**Health Insurance**								
No	Ref	Ref	Ref	Ref	Ref	Ref	Ref	Ref
Yes	**0.242(0.03-1.81)0.167**	**0.497(0.21-1.15)0.104**	**0.434(0.27-0.70)0.001**	**0.808(0.57-1.15)0.233**	**0.376(0.15-0.94)0.037**	1.926(0.61-6.06)0.262	**0.471(0.26-0.85)0.012**	**0.598(0.37-0.95)0.033**
**Family Size**								
1-2	Ref	Ref	Ref	Ref	Ref	Ref	Ref	Ref
3-4	**0.487(0.23-1.05)0.065**	**5.383(2.51-11.52)0.000**	**1.383(0.91-2.10)0.127**	**1.539(1.20-1.98)0.001**	**0.376(0.15-0.94)0.037**	2.089(0.55-7.90)0.227	0.87(0.49-1.54)0.631	0.978(0.66-1.45)0.911
5 or more	0.665(0.20-2.19)0.502	**15.272(0.22-0.94)0.000**	**1.769(0.70-4.45)0.226**	**3.846(2.50-5.92)0.000**	0.546(0.13-0.94)0.415	5.124(1.21-7.90)0.277	0.995(0.30-3.33)0.993	1.741(0.93-3.27)0.085
**Rota Virus Vaccination for Child**								
No	Ref	Ref	Ref	Ref		Ref		Ref
Yes	1.056(0.24-4.70)0.943	**14.238(6.03-33.62)0.001**	0.930(0.44-1.95)0.848	**2.781(1.81-4.8)0.000**		**9.517(2.88-31.47)0.026**		**3.037(1.69-5.47)0.000**
**Age of Child (months)**								
1-8	Ref	Ref	Ref	Ref	Ref	Ref	Ref	Ref
9-16	**6.203(2.48-15.53)0.000**	1.419(0.77-2.62)0.264	**3.019(1.52-6.00)0.002**	**1.815(1.32-2.50)0.000**	**8.458(2.95-24.22)0.000**	**2.495(1.15-5.40)0.020**	**2.339(1.06-5.18)0.036**	**3.468(1.69-5.47)0.000**
17-25	**14.108(3.28-60.73)0.00**	**4.561(2.30-9.05)0.000**	**3.214(1.56-6.62)0.002**	2.599(0.85-1.60)0.335	**11.403(2.460-52.84)0.002**	**10.225(3.62-28.84)0.000**	**2.558(1.10-6.03)0.029**	**5.070(3.05-8.44)0.000**
26-89	1	**7.498(410-13.71)0.000**	1.446(0.72-2.89)0.297	1.169(0.85-1.60)0.335	1	**11.203(3.90-32.10)0.000**	1.664(0.51-2.64)0.712	**3.627(2.14-6.14)0.000**
**Sex of Child**								
Female	Ref	Ref	Ref	Ref				Ref
Male	0.958(0.48-1.91)0.905	0.926(0.60-1.42)0.726	0.788(0.53-1.18)0.248	**0.840(0.66-1.06)0.147**				0.893(0.66-1.21)0.468
**Child received dewormer at 24 months and above**								
No	Ref	Ref	Ref	Ref		Ref		Ref
Yes	1.094(0.32-3.75)0.886	**2.186(1.34-3.56)0.002**	0.852(0.56-1.29)0.453	**0.422(0.33-0.54)0.000**		0.710(0.27-1.85)0.484		0.713(0.47-1.08)0.111
**Water source**								
Improved	Ref	Ref	Ref	Ref	Ref	Ref	Ref	Ref
Unimproved	**5.149(1.55-17.14)0.008**	**2.164(1.27-3.68)0.004**	**5.913(1.58-22.19)0.008**	**5.706(3.76-8.67)0.000**	**5.585(1.33-23.33)0.018**	0.907(0.42-1.94)0.802	**10.136(2.06-49.92)0.004**	**1.99(1.19-3.36)0.009**
**Latrine Usage in the house**								
No	Ref	Ref	Ref	Ref			Ref	
Yes	1.333(0.49-3.61)0.572	1.259(0.53-3.00)0.603	**0.378(0.24-0.59)0.000**	1.124(0.81-1.56)0.487			**0.392(0.21-0.73)0.003**	
**Domestic animal present in the house**								
No	Ref	Ref	Ref	Ref	Ref	Ref	Ref	
Yes	**1.791(0.87-3.69)0.114**	**15(4.26-52.87)0.000**	**1.843(0.72-4.70)0.200**	0.889(0.62-1.28)0.525	1.657(0.68-4.01)0.263	**7.535(1.10-51.50)0.039**	1.010(0.33-3.05)0.985	
**Washing hands before breastfeeding/cooking**								
No	Ref	Ref	Ref	Ref			Ref	
Yes	0.543(0.16-1.83)0.325	0.749(0.15-3.66)0.721	**0.419(0.27-0.65)0.00**	1.086(0.79-1.50)0.613			0.603(0.33-1.08)0.087	
**Availability of handwashing facilities in the house**								
No	Ref	Ref	Ref	Ref	Ref	Ref	Ref	Ref
Yes	**0.234(0.11-0.50)0.000**	**0.595(0.39-0.92)0.018**	**0.485(0.32-,0.73)0.001**	**0.413(0.33-0.53)0.000**	**0.329(0.12-0.87)0.025**	0.888(0.48-1.66)0.710	0.765(0.43-1.37)0.371	**0.533(0.38-0.74)0.000**
**Handwashing with soap help prevent diarrhoea infection**								
No	Ref	Ref	Ref	Ref	Ref		Ref	Ref
Yes	**2.930(0.78-10.92)0.109**	1.321(0.12-14.72)0.821	**2.346(1.44-3.81)0.001**	**5.900(4.01-8.68)0.000**	4.963(0.72-34.23)0.104		**3.059(1.45-6.47)0.003**	**3.441(1.81-6.54)0.000**
**Diarrhoea transmission through contaminated water**								
No	Ref	Ref	Ref	Ref				Ref
Yes	0.672(0.15-2.93)0.597	0.523(0.06-4.52)0.557	1.216(0.80-1.85)0.361	**2.940(2.15-4.02)0.000**				1.141(0.61-2.15)0.681
**Diarrhoea transmission through contaminated food**								
No	Ref	Ref	Ref	Ref			Ref	Ref
Yes	0.748(0.17-3.26)0.700	1.056(0.20-5.52)0.948	**1.616(1.01-2.58)0.044**	**3.106(2.20-4.38)0.000**			1.207(0.58-2.51)0.615	0.825(0.44-3.59)0.662

Bold values indicate statistical significance at p < 0.05 for multivariate and p < 0.25 for bivariate regression. COR = Crude Odds Ratio; AOR = Adjusted Odds Ratio; CI = Confidence Interval. “Ref” denotes the reference category for each categorical variable; child age is in months and caregiver/guardian age is in years; family size is number of household members; district was used to stratify columns and was also entered directly as a covariate in the pooled multivariable model.

## Discussion

Diarrhoea remains a leading cause of morbidity and mortality among children under five in sub-Saharan Africa, including Ghana, where it consistently ranks among the top causes of childhood illness. Despite several national and subnational studies, evidence on the comparative burden and determinants of diarrhoea across geographically and sociodemographically diverse districts in Ghana remains limited. This study addressed that gap by pooling data from three facility-based on cross-sectional surveys conducted in Ga West, Gomoa East, and Jirapa districts, representing urban, rural, and mixed residential settings, to estimate prevalence and identify independent determinants of diarrhoea among children under five. Generating district-specific evidence is important given Ghana’s well-documented geographic heterogeneity in diarrhoeal disease burden, which demands context-sensitive rather than uniform national responses.

The pooled prevalence of diarrhoea was 66.35%, with substantial district-level variation: Ga West recorded the highest burden (91.70%), followed by Gomoa East (72.25%) and Jirapa (34.83%). Child age, unimproved water source, and WASH infrastructure were the most consistent determinants across all three districts. Tertiary caregiver education, availability of handwashing facilities, and NHIS enrolment were independently protective in the pooled model even after adjusting for district, whereas deworming, laterine use, household size, and rural residence, which appeared protective or hazardous in the earlier unadjusted pooled estimates or within specific districts, did not retain independent significance in the pooled model. District itself emerged as one of the strongest independent predictors in the pooled model, with children in Gomoa East and Jirapa having markedly lower odds of diarrhoea than those in Ga West, net of all measured individual- and household-level factors. Ethnicity-based differences further highlight the role of sociocultural and contextual factors in shaping disease burden across the three study sites. Although the three district-level datasets were collected independently using the same data collection instrument, potential heterogeneity in data collection procedures and variations in local contextual factors may still have influenced the comparability of the pooled estimates, despite efforts to harmonize the datasets.

The persistence of a strong, independent district effect after adjusting for all measured individual- and household-level covariates is an important finding. Gomoa East and Jirapa’s markedly lower odds of diarrhoea relative to Ga West, even after accounting for age, education, water source, sanitation, and other measured factors, indicate that unmeasured district-level characteristics, such as differences in water infrastructure quality, environmental sanitation coverage, health system capacity, or local disease transmission dynamics, are likely driving a substantial portion of the observed variation. This is consistent with other Ghanaian evidence of persistent sub-national geographic heterogeneity in diarrhoeal disease: Duah et al. used spatial mapping to demonstrate marked district-level clustering of comorbid diarrhoea and anaemia among Ghanaian children that was not fully explained by individual-level covariates [14], while Tetteh et al.’s district-level surveillance analysis in the Jasikan District documented substantial year-on-year and sub-district variation in diarrhoea morbidity that pointed to local environmental and health-system drivers rather than purely individual risk factors [15].

The overall pooled prevalence of 66.35% considerably exceeds estimates from national surveys, including the 11.7% and 13% reported in the 2014 and 2022 Ghana Demographic and Health Surveys (GDHS), respectively [4,16]. This disparity is primarily attributable to the facility-based design and six-month recall period employed in this study, which captures a broader window of illness than the two-week recall used in DHS surveys and is more likely to enrol children with recent illness histories given health facility attendance. District-level variation is consistent with the spatio-temporal heterogeneity in childhood diarrhoea documented across Ghana over three decades [6,17]. Ga West’s high prevalence, despite its urban setting, challenges the assumption that urban residence is uniformly protective, supporting findings by Kumi-Kyereme and Amo-Adjei that urban poor households bear a disproportionate diarrhoeal burden [18]. The prevalence in Gomoa East (72.25%) is notably higher than the 36% reported in selected coastal communities in Ghana by Bandoh and his colleagues in 2024 [5], again reflecting methodological and recall period differences. The declining national trend from 26% in 1988 to 13% in 2022 cited by Kombat & Kushitor is encouraging but suggests that facility-level and district-level burdens may remain substantially higher than population surveys revealed [6].

Child age was the most consistent and statistically significant predictor of diarrhoea across all three districts and in the pooled analysis, with children aged 9–16, 17–25, and 26–89 months at significantly elevated risk compared to infants aged 1–8 months. This pattern aligns with evidence from multiple Ghana-based studies. Kombat et al. reported that children aged 6–35 months had the highest diarrhoea prevalence in the 2014 GDHS [16], while Apanga and Kumbeni, using nationally representative Multiple Indicator Cluster Surveys (MICS) data, found that children aged 6–23 months had over twice the odds of diarrhoea compared to infants aged 0–5 months [19]. Across East Africa, Tareke et al. similarly reported that children aged 7–12 months had 3.10-fold higher odds of diarrhoea [20]. The biological basis for this age-related vulnerability is well-established: infants below six months benefit from the immunological protection afforded by exclusive breastfeeding, whereas older infants transitioning to complementary feeding are exposed to potentially contaminated foods and water [21]. As children become increasingly mobile and engage in exploratory oral behaviour, contact with environmental pathogens intensifies. The markedly elevated odds in children aged 17–25 months observed across all districts suggest that the weaning period represents a critical window for targeted preventive interventions.

Access to safe WASH was independently associated with diarrhoea risk across all three districts. Unimproved water source significantly increased odds of diarrhoea in the pooled analysis, Ga West, and Jirapa, with effects ranging from over twofold to tenfold higher odds. This is consistent with Afrifa-Anane et al., who reported that improved water source reduced the likelihood of comorbid diarrhoea and acute respiratory infection in Ghana [22], and with Tetteh et al., who demonstrated that hygienic disposal of children’s stools is closely linked to latrine availability and significantly reduced diarrhoeal risk in pooled GDHS data [23]. Availability of household handwashing facilities was independently protective in the pooled analysis and in Ga West, corroborating findings by Pienaah et al. that physical WASH infrastructure remains a critical structural determinant of childhood diarrhoea in Ghana [24]. Latrine use was protective in Jirapa, where baseline sanitation coverage was lower, consistent with the broader African evidence synthesised by Azanaw et al. in 2024 [2]. Paradoxically, caregiver knowledge that handwashing with soap prevents diarrhoea was associated with significantly higher odds of diarrhoea in both the pooled analysis and Jirapa. This finding likely reflects a knowledge–practice gap, where awareness of preventive behaviours does not translate into consistent practice, a phenomenon widely attributed to structural and economic barriers that impede behaviour change in low-resource settings [2,24]. This underscores that health education alone, without accompanying structural WASH enablers, is insufficient to reduce diarrhoeal burden. This interpretation should, however, be regarded as one plausible explanation among several: reverse causation is also possible, in that caregivers of children who have already experienced diarrhoea may have received more health education on handwashing during clinic visits.

Tertiary caregiver education was independently protective in the pooled analysis (66.7% lower odds) and in Gomoa East. This is consistent with Apanga and Kumbeni, who found that children of mothers with college or higher education had 59% lower odds of diarrhoea compared to those with no formal education [19], and with Tampah-Naah, who similarly identified secondary or higher maternal education as protective [21]. Higher education likely equips caregivers with health literacy and economic resources to practise safer hygiene and feeding behaviours regardless of where they live. Health insurance (NHIS) enrolment emerged protective in the pooled analysis (40.2% lower odds), as well as in Ga West and Jirapa specifically, suggesting that removing financial barriers to care promotes timely treatment-seeking and reduces disease severity across different geographic contexts. Rotavirus vaccination remained associated with higher odds of diarrhoea in the pooled analysis after adjusting for district (AOR: 3.04) and markedly so in Gomoa East (AOR: 9.517); as before, this is unlikely to reflect a true biological effect, and more plausibly reflects selection of vaccinated children into facility-based care, a form of selection bias. This contrasts with findings by Bandoh et al., who reported that fully vaccinated children had 32% lower diarrhoea prevalence in coastal communities using a community-based design, reinforcing the interpretive caution warranted by this result [5].

### Limitations

This study has several limitations. The facility-based design means findings may not fully represent the broader under-five population, as children with mild or self-managed diarrhoea are less likely to present at health facilities. The six-month recall period, while providing a broader epidemiological window, is susceptible to recall bias and departs from the standard two-week WHO surveillance window, limiting direct comparability with national survey estimates. The pooled analysis used equal weights across districts, given the equal sample sizes; however, this may not fully account for the different population sizes each district represents. Additionally, unmeasured confounders such as child nutritional status, microbiological water quality, and food hygiene practices could not be accounted for. Several further sources of potential bias should be noted. Diarrhoea status, water source, and hygiene practices were all self-reported by caregivers, which may have introduced recall bias and social-desirability bias (for example, over-reporting of handwashing and safe-water practices). Despite these limitations, the multi-district pooled design, adequate sample size, and use of multivariable logistic regression with adjustment for relevant confounders strengthen the validity and policy relevance of the findings.

## Conclusion

Diarrhoea among children under five remains a substantial public health burden across the three study districts. District itself is an independent determinant of that burden: even after adjusting for measured individual and household-level factors, children in Gomoa East and Jirapa had significantly lower odds of diarrhoea than those in Ga West. Child age, unimproved water source, poor handwashing infrastructure, were key modifiable risk factors while tertiary caregiver education, NHIS enrolment, and handwashing facility availability remained protective. The observed knowledge–practice gap around handwashing suggests that education alone is insufficient without accompanying structural WASH improvements.

District health authorities should prioritise targeted interventions for children aged 9–25 months, scale up NHIS enrolment, and invest in WASH infrastructure, particularly in high-burden peri-urban settings. Future research should adopt community-based designs with objective WASH measurements to strengthen causal inference. Diarrhoea remains a serious health challenge among children under five in sub-Saharan Africa. It continues to contribute to preventable loss of life each year, underscoring the need for sustained investment in prevention. Important gaps that future research should address include community-based (rather than purely facility-based) sampling to reduce selection bias, and objective measurement of water quality and hygiene practices to reduce reliance on self-report; addressing these gaps will help clarify which associations reported here reflect true determinants versus study-design artefacts, and will support the design of effective mitigation strategies.

## Supporting information

S1 File TTI data. (ZIP)

## References

[1] World Health Organization. Diarrhoeal disease. WHO; 2024. https://www.who.int/news-room/fact-sheets/detail/diarrhoeal-disease

[2] Azanaw J, Malede A, Yalew HF, Worede EA. Determinants of diarrhoeal diseases among under-five children in Africa (2013-2023): A comprehensive systematic review highlighting geographic variances, socioeconomic influences, and environmental factors. BMC Public Health. 2024;24(1):2399. doi: 10.1186/s12889-024-19962-0 39232730 PMC11373296

[3] Yirsaw BG, Mezen MK, Belete AK, Asfaw FF, Yerie ST, Nega AK, et al. Prevalence of diarrheal disease and its determinants among children under five in East Africa: Systematic review and meta-analysis. BMC Infect Dis. 2025;25(1):1262. doi: 10.1186/s12879-025-11595-x 41068656 PMC12512649

[4] Ghana Statistical Service, ICF. Ghana Demographic and Health Survey 2022: Key Indicators Report. Accra: Ghana Statistical Service and ICF. 2023.

[5] Bandoh DA, Dwomoh D, Yirenya-Tawiah D, Kenu E, Dzodzomenyo M. Prevalence and correlates of diarrhoea among children under five in selected coastal communities in Ghana. J Health Popul Nutr. 2024;43(1):95. doi: 10.1186/s41043-024-00582-8 38926857 PMC11210189

[6] Kombat MY, Kushitor SB. Trends and determinants of childhood diarrhea in Ghana: Evidence from the Ghana Demographic and Health Survey (1988–2022). Discov Public Health. 2025;22:340.

[7] Mosley WH, Chen LC. An analytical framework for the study of child survival in developing countries. Popul Dev Rev. 1984;10(Suppl):25–45. PMC2572391 12756980

[8] Ghana Statistical Service. 2010 Population and Housing Census: District Analytical Report – Jirapa District. Accra: Ghana Statistical Service; 2014.

[9] Ghana Statistical Service. 2010 Population and Housing Census: District Analytical Report – Ga West Municipal. Accra: Ghana Statistical Service; 2014.

[10] Ghana Statistical Service. 2010 Population and Housing Census: District Analytical Report – Gomoa East District. Accra: Ghana Statistical Service; 2014.

[11] Ghana Statistical Service. 2021 Population and Housing Census: General Report Volume 3A, Population of Administrative Units. Accra: Ghana Statistical Service; 2021.

[12] Cochran WG. Sampling Techniques. 3rd ed. New York: John Wiley & Sons; 1977.

[13] World Health Organization, United Nations Children’s Fund. Progress on drinking water, sanitation and hygiene: 2017 update and SDG baselines. Geneva: WHO Press; 2017.

[14] Duah HO, Amankwa CE, Adomako I, Owusu B, Agbadi P. Comorbid patterns of anaemia and diarrhoea among children aged under 5 years in Ghana: A multivariate complex sample logistic regression analysis and spatial mapping visualisation. Int Health. 2021;13(6):562–72. doi: 10.1093/inthealth/ihaa099 33339041 PMC8643461

[15] Tetteh J, Takramah WK, Ayanore MA, Ayanore AA, Bisung E, Alamu J. Trends for diarrhea morbidity in the Jasikan District of Ghana: Estimates from district level diarrhea surveillance data, 2012–2016. J Trop Med. 2018;2018:4863607. doi: 10.1155/2018/4863607 PMC6198541 30402113

[16] Kombat MY, Kushitor SB, Sutherland EK, Boateng MO, Manortey S. Prevalence and predictors of diarrhea among children under five in Ghana. BMC Public Health. 2024;24(1):154. doi: 10.1186/s12889-023-17575-7 38212722 PMC10782682

[17] Afitiri A-R, Armah FA, Ekumah B, Nyieku FE, Yawson DO, Odoi JO. Cumulative effects of environmental factors on household childhood diarrhoea in Ghana. Water Practice and Technology. 2020;15(4):1032–49. doi: 10.2166/wpt.2020.083

[18] Kumi-Kyereme A, Amo-Adjei J. Household wealth, residential status and the incidence of diarrhoea among children under-five years in Ghana. J Epidemiol Glob Health. 2016;6(3):131–40. doi: 10.1016/j.jegh.2015.05.001 26070430 PMC7320473

[19] Apanga PA, Kumbeni MT. Factors associated with diarrhoea and acute respiratory infection in children under-5 years old in Ghana: An analysis of a national cross-sectional survey. BMC Pediatr. 2021;21(1):78. doi: 10.1186/s12887-021-02546-x 33581716 PMC7881472

[20] Tareke AA, Enyew EB, Takele BA. Pooled prevalence and associated factors of diarrhea among under-five years children in East Africa: A multilevel logistic regression analysis. PLoS One. 2022;17(4):e0264559. doi: 10.1371/journal.pone.0264559 35421129 PMC9009646

[21] Tampah-Naah AM. Maternal and child level factors associated with childhood (0-23 months) diarrhoea in Ghana: A pooled analysis of national representative datasets. Ghana J Dev Stud. 2019;16(2):157. doi: 10.4314/gjds.v16i2.8

[22] Afrifa-Anane GF, Kyei-Arthur F, Agyekum MW, Afrifa-Anane EK. Factors associated with comorbidity of diarrhoea and acute respiratory infections among children under five years in Ghana. PLoS One. 2022;17(7):e0271685. doi: 10.1371/journal.pone.0271685 35862358 PMC9302777

[23] Tetteh J, Adomako I, Udofia EA, Yarney E, Quansah H, Yawson AO. Hygienic disposal of stools and risk of diarrheal episodes among children aged under two years: evidence from the Ghana Demographic Health Survey, 2003–2014. PLoS One. 2022;17(4):e0266681. doi: 10.1371/journal.pone.0266681 PMC8989342 35390094

[24] Pienaah CKA, Yoshida Y, Saaka SA, Ategeeng FN, Luginaah I. Determinants of diarrhea prevalence among children under 5 years in semi-arid Ghana. Journal of Water, Sanitation and Hygiene for Development. 2024;14(7):583–94. doi: 10.2166/washdev.2024.092

